# Genome sequences of hepatitis E virus genotype 1e from an outbreak in the Central African Republic, 2018

**DOI:** 10.1128/mra.00932-26

**Published:** 2026-09-30

**Authors:** Marina Prisca de Marguerite Nombot-Yazenguet, Amal Chaghouri, Narcisse Patrice Joseph Komas, Anne-Marie Roque-Afonso

**Affiliations:** 1 Viral Hepatitis Laboratory, Institut Pasteur de Bangui 673899, Bangui, Central African Republic; 2 Université Paris-Saclay, Inserm U1993, AP-HP Hôpital Paul Brousse Virology, Villejuif, France; DOE Joint Genome Institute, Berkeley, California, USA

**Keywords:** hepatitis E virus, Central African Republic

## Abstract

The hepatitis E virus (HEV) causes major outbreaks in sub-Saharan Africa. Here, we present four genome sequences of HEV-1e from the 2018 outbreak in the Central African Republic. Despite high identity with known African isolates, these sequences constitute a new cluster and suggest local transmission and diversification.

## ANNOUNCEMENT

The hepatitis E virus (HEV) is a major cause of acute viral hepatitis worldwide (1). This small, positive-sense RNA virus belongs to the *Hepeviridae* family. The species *Paslahepevirus balayani*, which comprises eight genotypes, accounts for most of the viruses responsible for human infections (2). HEV-3 and HEV-4 are zoonotic, leading to sporadic cases in industrialized countries. In contrast, HEV-1 and HEV-2 infect only humans and are transmitted via the fecal-oral route in low- and middle-income countries (3). HEV strains linked to outbreaks and sporadic cases in sub-Saharan Africa have so far been classified mainly as subtype 1e (HEV-1e). The first genome was recovered from the 1983 Chad’s outbreak (4). Related strains were subsequently observed during outbreaks in Niger in 2017 (5) and Nigeria (6). Other sequences were reported from Niger in 2021–2022 (6) and from icteric patients with suspected yellow fever in Cameroon in 2022 (7). We previously described an HEV outbreak in 2018–2019 in the Bocaranga and Koui health districts of the Central African Republic (CAR), where HEV-1e was identified by partial ORF2 sequencing (8).

Four serum samples collected during this study and with sufficient sample volume were used for whole-genome sequencing with a viral capture enrichment approach (Viral Surveillance Panel, Illumina, San Diego, CA, USA). Nucleic acids were extracted from 200 µL of serum using the QIAsymphony DSP Virus/Pathogen Mini Kit (QIAGEN, Hilden, Germany), with an elution volume of 90 µL. Extracts were processed with the Illumina RNA Prep with Enrichment, (L) Tagmentation kit for complementary DNA and double-stranded DNA synthesis, and library generation by on-bead tagmentation, which simultaneously fragments DNA and incorporates sequencing adapters. Indexed libraries were subsequently hybridized with the viral probe set for enrichment. After overnight incubation, probe-target hybrids were captured with streptavidin-coated magnetic beads and amplified by post-capture PCR to generate enriched libraries. Sequencing was performed on a MiSeq instrument configured for 2 × 150 base pairs paired-end reads using a MiSeq Reagent Kit v3. A custom bioinformatic pipeline was developed by MOABI AP-HP (https://moabi-aphp.github.io/index.html). We used Cutadapt (v.2.3; options: -a CTGTCTCTTATACACATCT -A CTGTCTCTTATACACATCT) to trim raw reads of adapter sequences and BWA-MEM (v.0.7.15; option: -M) for mapping to reference GenBank number L08816. Consensus sequences were generated with Samtools (v1.19.2): one under default parameters and one ambiguous consensus incorporating IUPAC degenerate codes (option: --ambig). Quality control (QC) was performed at each step: read quality was evaluated using FastQC (v0.11.9-2), while alignment metrics and target coverage were computed using Samtools flagstat and coverage utilities. All QC outputs were aggregated into a unified report using MultiQC (v1.21).

**Fig 1:**
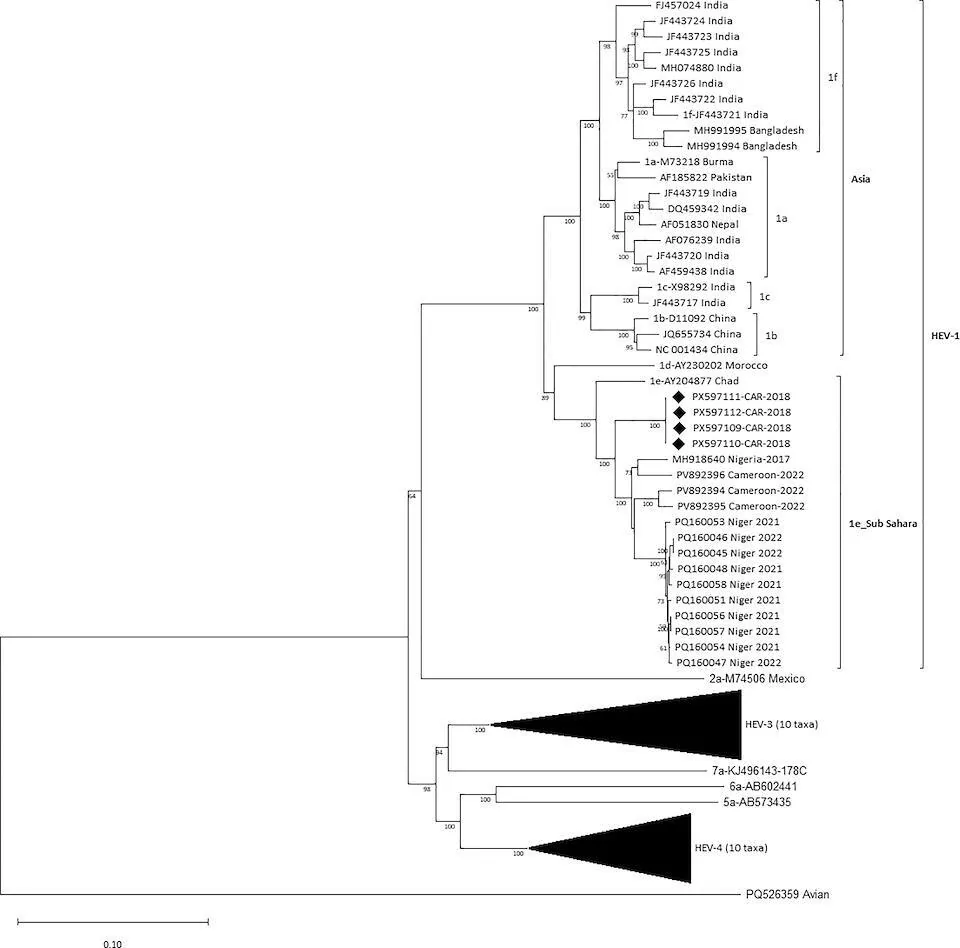
Phylogenetic tree based on genome sequences of proposed reference strains, labeled with subtype designation (9), accession number, and country of origin, together with HEV-1 sequences available in GenBank. Sub-Saharan and Asian HEV-1 sequences, as well as HEV-1 subtypes, are indicated by brackets. HEV-1e sequences identified in CAR are indicated by ◆. Multiple sequence alignment was performed using ClustalX2, followed by phylogenetic analysis including 70 taxa in MEGA12 using the neighbor-joining method, with distances calculated by the Kimura 2-parameter method. Only bootstrap test values >90% (500 replicates) are shown next to the branches. The complete deletion option was applied to eliminate positions containing gaps and missing data, resulting in a final data set comprising 6,383 positions. Reference sequences with accession numbers were 1a, M73218 (Burma); 1b, D11092 (China); 1c, X98292 (India); 1d, AY230202 (Morocco); 1e, AY204877 (Chad); 1f, JF443721 (India); 2a, M74506 (Mexico); 3a, AF082843; 3b, AP003430; 3c, FJ705359; 3e, AB248521; 3f, AB369687; 3h, JQ013794; 3g, AF455784; 3j, AY115488; 3i, FJ998008; 3ra, FJ906895; 4, AB369688; 4a, AB197673; 4b, DQ279091; 4c, AB074915; 4d, AJ272108; 4e, AY723745; 4f, AB220974; 4g**,** AB108537; 4h, GU119961; 4i-DQ450072; 5a, AB573435; 6a, AB602441; 7a, KJ496143; and Avian, PQ526359.

The four sequences obtained from the 2018 outbreak in the CAR (Table 1) were highly related (average percent identity: >99.9) and formed a distinct cluster within HEV-1e sub-Saharan sequences (Fig. 1). These sequences broaden the known genetic diversity of HEV-1e in sub-Saharan Africa. Their clustering is consistent with local transmission and diversification and improves our understanding of HEV phylogeography in Africa.

**TABLE 1:** Characteristics of the 4 HEV genomes identified^*a*^

GenBank_accession	SRA_accession	Year of collection	Country of sampling	Age (years) sex	HEV RNA (log IU/mL)	ALT (IU/mL)	Total reads (million)	Aligned reads	Coverage (%)	Average depth of coverage	Genome length	G + C (%)
PX597109	SRX34562779	2018	CAR	27, female	6.24	79	5.18	12,280	97.6	232.2×	7,159	58.1
PX597110	SRX34562780	2018	CAR	26, female	5.83	288	4.34	24,316	97.8	470.9×	7,192	57.66
PX597111	SRX34562781	2018	CAR	53, male	5.38	32	4.23	5,425	97.0	103.7×	7,156	57.75
PX597112	SRX34562782	2018	CAR	28, female	5.82	682	4.27	2,815	97.0	54.2×	7,116	57.85

^*a*^ HEV viral load was determined by using the Realstar HEV RT-PCR Kit (Altona Diagnostics, Hamburg, Germany).

## Data Availability

The near complete genome sequences from CAR have GenBank under accession numbers PX597109, PX597110, PX597111, and PX597112. The raw reads have been deposited in the NCBI Sequence Read Archive (SRA) under accessions numbers SRX34562779, SRX34562780, SRX34562781, and SRX34562782, and BioProject number PRJNA1504041.
